# Role of Transition Metals in Pt Alloy Catalysts for the Oxygen Reduction Reaction

**DOI:** 10.1021/acscatal.3c03321

**Published:** 2023-11-03

**Authors:** Chaewon Lim, Alasdair R. Fairhurst, Benjamin J. Ransom, Dominik Haering, Vojislav R. Stamenkovic

**Affiliations:** † Department of Chemical & Biomolecular Engineering, University of California, Irvine, California 92697, United States; ‡ HORIBA Institute for Mobility and Connectivity, University of California, Irvine, California 92697, United States; § Department of Chemistry, University of California, Irvine, California 92697, United States

**Keywords:** electrocatalysis, oxygen reduction reaction, platinum alloy, transition metal, ligand effect, strain effect, surface science, spectroscopy

## Abstract

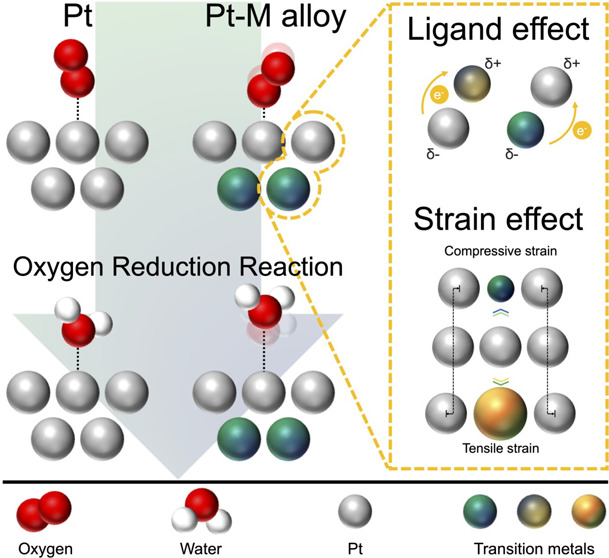

In pursuit of higher activity and stability of electrocatalysts toward the oxygen reduction reaction, it has become standard practice to alloy platinum in various structural configurations. Transition metals have been extensively studied for their ability to tune catalyst functionality through strain, ligand, and ensemble effects. The origin of these effects and potential for synergistic application in practical materials have been the subject of many theoretical and experimental analyses in recent years. Here, a comprehensive overview of these phenomena is provided regarding the impact on reaction mechanisms and kinetics through combined experimental and theoretical approaches. Experimental approaches to electrocatalysis are discussed.

## 1 Introduction

The polymer electrolyte membrane fuel cell (PEMFC) is a developing electrochemical technology with the potential to revolutionize the transportation sector. The power output and longevity of commercial PEMFCs are linked to the performance of the cathode, where the oxygen reduction reaction (ORR) occurs with intrinsic challenges in long-term activity and stability.^1,2^ The ORR is particularly critical in determining the overall energy conversion efficiency of PEMFCs due to inherently hindered reaction kinetics.^3−5^ For those reasons, the development of high-performance ORR catalysts has been pivotal in the ongoing deployment of commercial PEMFC technology.

Platinum (Pt) and its alloys show high activity and stability as catalysts toward the ORR, both of which are required for application in the acidic PEMFC operating environment; however, widespread use of PGM catalysts is limited by the high cost and scarcity of raw materials.^5,6^ Alloys of Pt with early transition metals have been adopted as a reliable solution for practical applications,^7^ as they can have superior stability and catalytic activity in comparison to bare Pt. Ligand,^8^ strain,^9^ and ensemble^10^ effects are recognized as mechanisms by which activity can be increased and activation overpotential reduced through what has become known as the “materials-by-design” strategy.^3,11^

The ligand effect refers to the influence of a foreign near-surface metal on the electronic structure of the host metal and subsequent changes in catalyst–adsorbate interactions.^12−14^ It is closely related to the strain effect, which equally influences catalyst activity through electronic structure modifications but is derived from the difference in atomic size of foreign and host metals, leading to compression or expansion of the surface atomic structure.^15,16^ The ensemble effect is related to the arrangement of atoms or small groups of atoms (ensembles) on the catalyst surface. The same term is often used to describe the catalytic effect of dissimilar elements or distinct arrangements of the same element on reaction kinetics.^17^ The modified physicochemical properties of alloy catalysts are the result of synergistic interplay between these phenomena, affecting the binding strength of adsorbed species including reactants, reaction intermediates, products, and even spectators.^18,19^

Structure–activity–stability relationships at the atomic level have been unraveled by advanced surface specific ultrahigh-vacuum (UHV) methods: high-resolution photoelectron spectroscopy, quantitative low-energy electron diffraction (LEED),^20^ low-energy ion scattering (LEIS),^21^ and ultraviolet photoelectron spectroscopy (UPS),^13^ in combination with rotating-disk electrode (RDE), *in situ* and *operando* transmission electron microscopy (TEM),^22^ scanning tunneling microscopy (STM),^23^ and inductively coupled plasma–mass spectroscopy (ICP-MS).^24^ Vibrational spectroscopic techniques, such as Raman and infrared (IR) spectroscopy, detect vibrational profiles of reaction intermediates, enabling indirect characterization of the electrocatalyst structure at the molecular level.^25^ In addition, density functional theory (DFT) calculations have been an invaluable tool to bridge theory and experiment.^26,27^ Understanding atomic level relationships provides the foundation for design of new synthesis and evaluation processes leading to the development of next-generation electrocatalysts.

In recent years, there have been numerous attempts to produce high-performance ORR catalysts through alloying transition metals with Pt; nevertheless, the particular contribution of the less noble metal in the reaction remains unclear. In this review, we revisit the role of transition metals in Pt–M alloy systems for ORR structure–activity–stability relationships in acidic media through the analysis of ligand, strain, and ensemble effects. The current understanding of these physicochemical effects is covered from both theoretical and state-of-the-art experimental perspectives, with an additional focus on *operando* experimental evaluation.

## 2 ORR Reaction Mechanism on Bimetallic Surfaces

### 2.1 Oxygen Reduction Reaction Pathways

**Figure 1:**
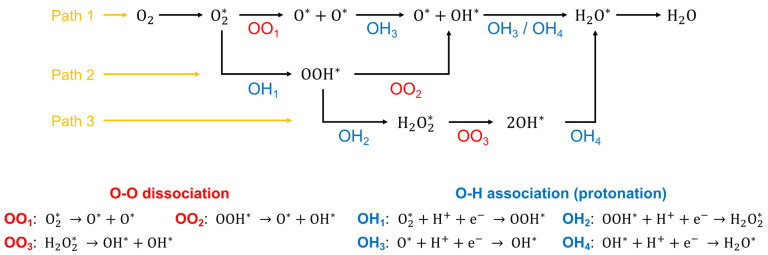
Three possible pathways, including three O–O dissociation (OO_*x*_) and four protonation (OH_*x*_) steps, for the ORR in acidic media. Modified and reproduced with permission from ref (28), 2008, Elsevier.

Mechanistic studies on the ORR have been conducted over the past half-century to explore alternative materials that can exhibit performance at the same or higher level than bare Pt.^29−38^ Based on rotating ring–disk electrode (RRDE) studies of model extended surfaces, three pathways with multiple elementary steps consisting of three O–O dissociation and four O–H association (protonation) reactions for the ORR have been proposed, illustrated in Figure 1.^39^ The asterisk (*) represents surface-active sites where the reaction intermediates can be adsorbed. Adsorbed *O_2_ is transformed into H_2_O by following one of the proposed reaction pathways, where self-adsorption of O_2_ molecules onto active sites is required to produce chemically adsorbed oxygen (*O_2_) without electron transfer.^40^ Paths 1 and 2 each are direct four-electron reactions and can be categorized as dissociative and associative mechanisms, respectively, either of which is desirable for fuel cell applications. Path 3 represents the indirect four-electron pathway, which is divided into the generation of an H_2_O_2_ intermediate on the surface through an initial two-electron transfer step and a subsequent two-electron protonation reaction.^28^ In each case, interactions between adsorbent and catalyst play a focal role in determining ORR performance.^40^ A strong oxygen binding energy causes a decrease in reaction rate by impeding the electron transfer and protonation (OH_*x*_ formation) step; in contrast, a weak binding energy contributes to slow O–O cleavage (OO_*x*_) and leads to inferior ORR kinetics.^41^ The oxygen binding energy is accordingly believed to be a good kinetic descriptor and is widely applied to DFT calculations to predict or explain catalytic performance.^3^

### 2.2 Combining Computational and Experimental Studies for Catalyst Design

**Figure 2:**
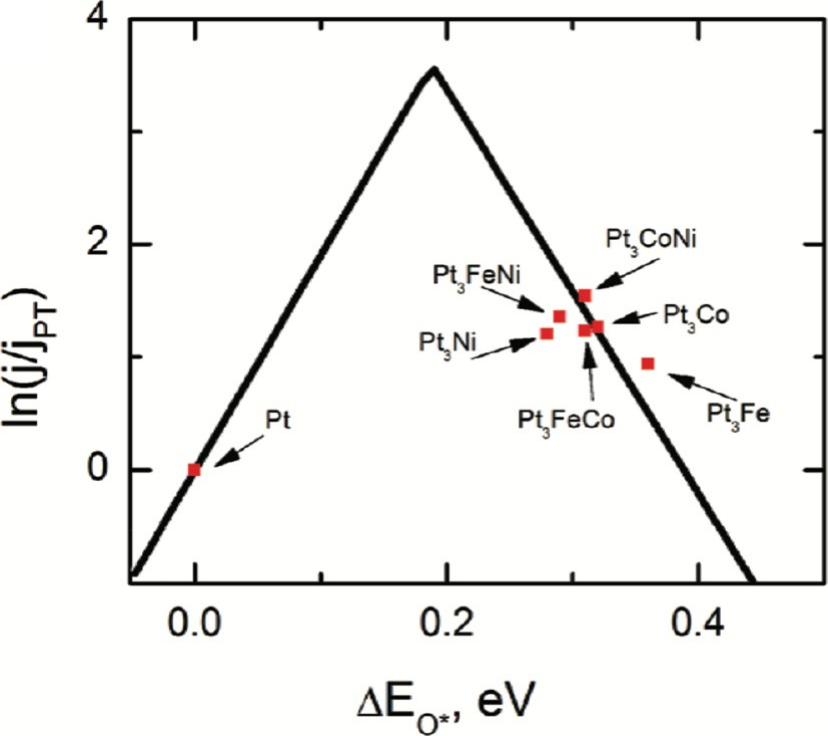
Volcano plot relationship of measured catalyst performance versus the DFT-calculated oxygen binding energy. The adsorption energy of oxygen (Δ*E*_O*_) is calculated relative to Pt(111); activities are scaled by values measured for Pt. Solid black activity lines are taken from DFT calculations used for Pt-based bimetallic catalysts. Reprinted with permission from ref (42), 2012, American Chemical Society.

Although previous studies have demonstrated that alloying Pt with transition metals improves catalytic activity,^2,4,14^ substantial challenges remain before wide deployment of PEMFCs can be achieved. In most modern theoretical analyses, the Sabatier principle is applied to relate the catalyst activity to the binding energy of reaction intermediates,^32^ where the balance between adsorption and desorption energy is critical to achieving high performance.^43^ Pt(111) is generally used as a model surface for both experimental and theoretical studies but has an oxygen binding energy 0.2 eV stronger than the optimum value for O–O bond cleavage.^44,45^ The Pt–M interaction changes the surface electronic structure and begins to explain the performance enhancement of Pt alloy catalysts through alterations in the adsorbate binding energy and is outlined by the volcano plot in Figure 2, where the apex of the volcano is 0.2 eV from Pt(111) binding energy and a number of results for Pt–M are shown.^46^

**Figure 3:**
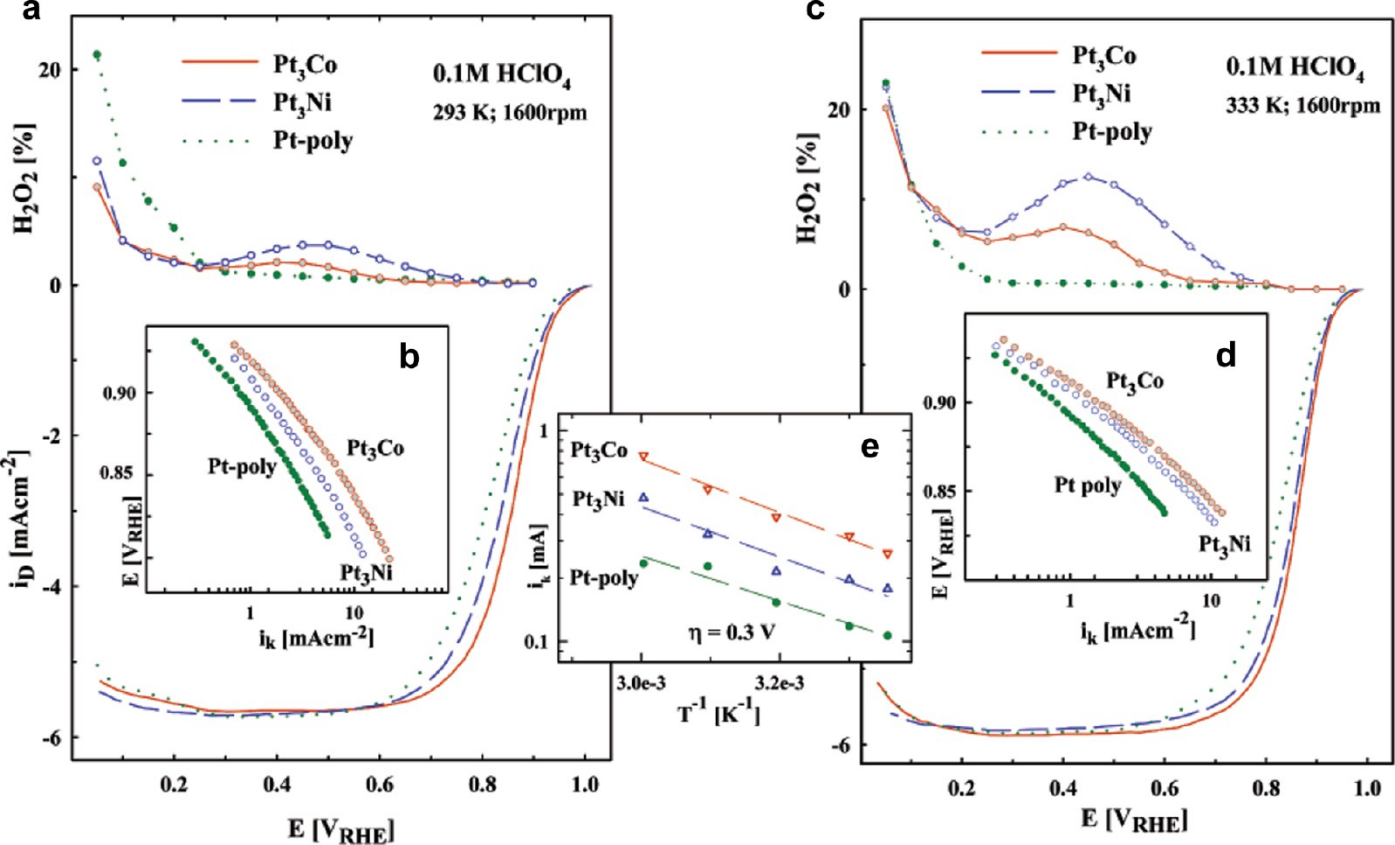
(a) Disk (*i*_D_) and ring (*i*_R_) currents (anodic sweep direction) during the ORR on mildly sputtered Pt, Pt_3_Co, and Pt_3_Ni in 0.1 M HClO_4_ at 293 K. (b) Tafel plots for all three surfaces at 293 K. (c) Disk (*i*_D_) and ring (*I*_R_) currents (anodic sweep direction) during the ORR on mildly sputtered Pt, Pt_3_Co, and Pt_3_Ni in 0.1 M HClO_4_ at 333 K. (d) Tafel plots for all three surfaces at 333 K. Conditions: sweep rate, 20 mV/s; ring potential, *E* = 1.15 V vs RHE; collection efficiency, *N* = 0.2. (e) Arrhenius plots at an overpotential of 0.3 V for the ORR on Pt, Pt_3_Co, and Pt_3_Ni electrodes. Reprinted with permission from ref (40), 2002, American Chemical Society.

Reaction mechanisms in electrocatalysis are affected by a number of variables which impact the validity of assumptions used in determining reaction pathways, particularly where the goal is to compare the performance of different materials.^47,48^ Marković et al. probed the effect of surface structure on the ORR mechanism experimentally by extracting kinetic parameters from RRDE measurements.^38^ The reaction order, Tafel slope, and apparent activation energy show similar values on Pt_3_Ni, Pt_3_Co, and Pt-poly surfaces, implying that the ORR mechanism on Pt–M alloys conforms to the same 4 e^–^ reduction pathway proposed for pure Pt (Figure 3).^40,49^ Assuming the same reaction mechanism applies in each case, DFT calculations can effectively predict catalyst performance from scaling relationships. Determination of the energy variable most relevant to catalytic activity for the particular reaction of interest is critical to ensuring models match the observed behavior; in most cases, the oxygen binding energy is used as a universal descriptor for the ORR.^44,45,50,51^ The application of a microkinetic model to the potential energy surface of a reaction is one such approach used to enable prediction of catalytic performance from adsorption and activation energies.^52^ The Brønsted–Evans–Polanyi (BEP)^53,54^ relation linearly relates the activation energy to enthalpy change from the initial to the final state in elementary reactions. More relevant to complex multistep elementary reactions, however, are transition state scaling (TSS)^55^ relations. As an extension of BEP and transition state theory, TSS relations have greater applicability to multistep processes and adequately describe the linear relationship between transition state energy and adsorption energy of reaction intermediates. The resulting adsorbate scaling relations^56^ simplify the complex expression of material-dependent catalytic activity to a single parameter through the linear relationship between the adsorption energy of each intermediate in a multistep reaction.^53,54^ In order to search for novel ORR catalysts, DFT calculations are applied to derive volcano plots based on optimizing the adsorbate binding energies.^27^ The three mechanisms observed experimentally form the basis of computational analysis through determination of adsorbate scaling relations to tune catalyst-adsorbate interactions i.e., ligand strain and ensemble effects.^16,18,51,57,58^

**Figure 4:**
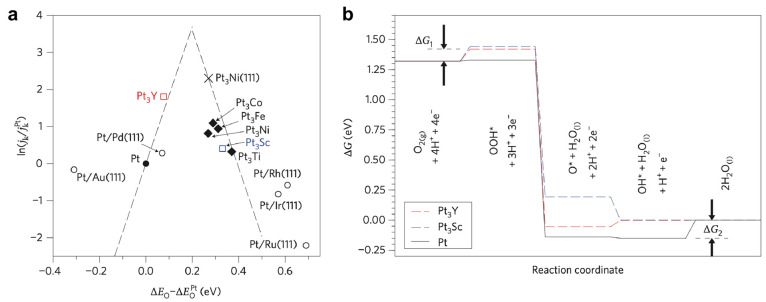
(a) Volcano plot for the oxygen reduction reaction on Pt-based transition-metal alloys as a function of oxygen binding energy. (b) Free-energy diagrams for the polycrystalline bulk Pt_3_Y and Pt_3_Sc catalysts. Reprinted with permission from ref (59), 2009, Springer.

Greeley et al.^59^ summarized experimentally determined ORR activities of various Pt-based catalysts from literature data and generated a theoretical volcano plot as a function of the oxygen adsorption energy relative to Pt (Figure 4a) to explain the relationship between oxygen adsorption energy and ORR activity. The dashed lines in Figure 4a are theoretical limits derived from TSS relations illustrating the excellent match between model and experimental data. The DFT free energy diagram (Figure 4b) is based on the associative reaction pathway and illustrates two encumbered reaction steps originating from the positive change in free energy (Δ*G*_1_ and Δ*G*_2_) at 0.9 V of *OOH formation and *OH desorption, respectively.^60^ The free energy change of each step is correlated to the stability of adsorbed intermediates (*OOH, *OH, and *O), characterized by Δ*E*_O_, which in turn are dependent on the electronic structure of the catalyst surface and the resulting catalyst–adsorbate interaction.^56^ The increase of Δ*E*_O_ (decrease of oxygen binding energy) destabilizes *OOH species on the surface and increases Δ*G*_1_. Simultaneously, the change in Δ*E*_O_ facilitates desorption of surface species (*OH or *O) and a decrease in Δ*G*_2_.^59^ Polycrystalline Pt_3_Y (Figure 4a)^56,60^ shows higher activity than Pt as a corollary of this effect, cementing the notion that introduction of transition metals modifies adsorption and desorption energies of reaction intermediates, rather than the reaction pathway.^56,60^

## 3 Ligand, Strain, and Ensemble Effects

### 3.1 Ligand Effects

**Figure 5:**
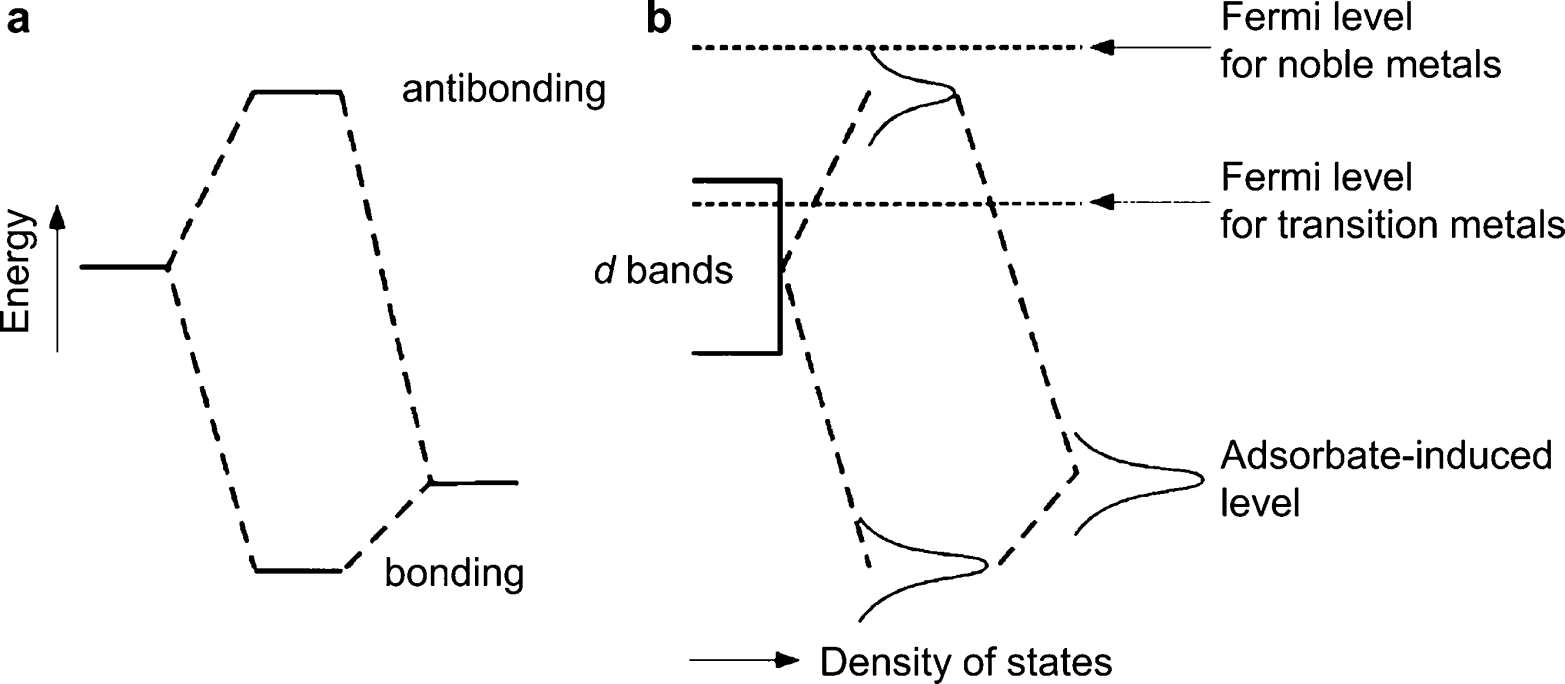
Schematic illustration of the interaction between two electronic states. The downshift of the bonding state is smaller than the upshift of the antibonding state because the overlap of the initial states gives rise to an energy cost related to the orthogonalization of the two states. Both the energy associated with the orthogonalization and the hybridization energy associated with the formation of bonding and antibonding states scale with the square of the coupling matrix element. (a) The simple case of two sharp atomic or molecular states. (b) The interaction between a state of an adsorbate outside a metal surface, which has been broadened out to a resonance owing to the interaction with the metal s-band, and the metal d-bands. Reprinted with permission from ref (61), 1995, Springer.

The performance enhancement of Pt alloy catalysts can be interpreted through the d-band theory of Hammer and Nørskov,^61^ which describes the correlation between the d-band center of a metallic surface and associated change in adsorption characteristics. The d-band center of a pure metal can be tuned by alloying, where the modified distribution of electrons in the d-band alters the adsorption characteristics of the surface. The ligand effect is defined by the contribution to activity of dissimilar atoms in the four topmost atomic layers, as the alloying element modifies the median energy level of the d-band.^18,19^ In the simplest case of hydrogen dissociation on Pt(111), the metal substrate d-band electrons bond with the s orbital electrons of the adsorbate.^27,50^ Stamenkovic et al.^14^ applied the same principles to the more complex ORR, where the p-orbital of the adsorbate overlaps with the d-band of the metal. When the d-band center reaches a characteristic level, higher energy electrons exist above the Fermi level, resulting in antibonding whereon unfilled antibonding states strengthen the interaction with adsorbates (Figure 5).^61^ By alloying Pt with transition metals and manipulating the location of the d-band center, it is possible to reduce the number of unfilled antibonding states and weaken surface interactions with adsorbates in pursuit of the optimal binding energy. The peak of the Sabatier volcano is defined by the resulting balance between adsorption and desorption of surface species and plays a critical role in determining the electrocatalytic performance for the ORR. On Pt surfaces in acidic media, the reaction rate is linked to the coverage of OH adsorbates on the surface through eq 1, where OH acts as a spectator species, blocking active sites for the reaction^62^
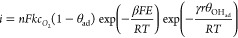
1where *n* is the number of electrons, *k* is the rate constant, *c*_*O*___2__ is the concentration of oxygen in the electrolyte, θ_ad_ is the surface coverage of blocking species including specifically adsorbing anions and OH_ad_ (θ_OH_ad__), β and γ are symmetry factors, *R* is the ideal gas constant, *T* is the temperature, and *r* is the rate of change of the apparent standard free energy of adsorption with coverage of adsorbing species.^62^ The adsorption energy of intermediates can be tuned by d-band manipulation to bring the adsorption energy closer to the optimal value, approximately 0.2 eV weaker than Pt(111), increasing surface site availability and thereby boosting activity in the process.^63^ The location of the d-band closely describes how the ligand effect will tune surface interactions and hence catalytic activity.^14,50,64,65^

**Figure 6:**
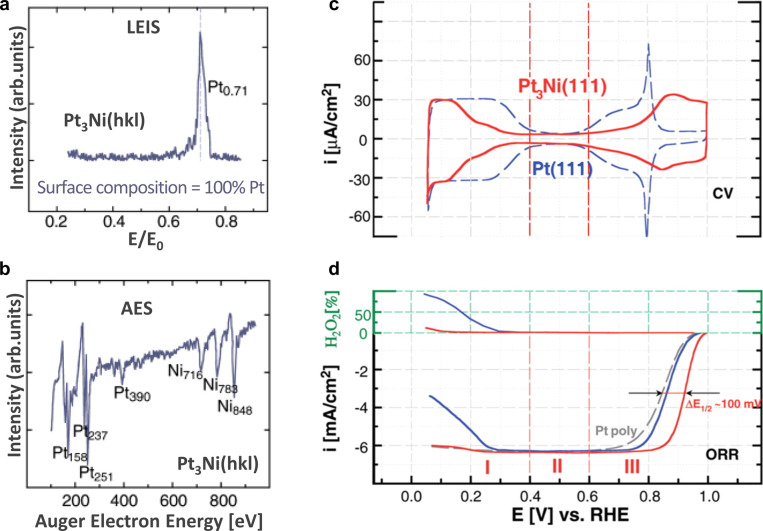
(a) LEIS and (b) AES spectra of Pt_3_Ni single crystals in UHV: *E*/*E*_0_, where *E* is the energy of scattered electrons and *E*_0_ is the energy of the incident ion beam. (c) Cyclic voltammetry in the designated potential region (red curve) as compared to the voltammetry obtained from the Pt(111) surface (blue curve). (d) Green scale referring to hydrogen peroxide production in the designated potential region and ORR currents measured on Pt_3_Ni(111) (red curve), Pt(111) (blue curve), and polycrystalline Pt (gray curve) surfaces. The arrows show the positive potential shift of 100 mV in electrode half-potential (Δ*E*_1/2_) between ORR polarization curves measured on Pt-poly and Pt_3_Ni(111) surfaces. I, II, and III represent potential regions of H_upd_ adsorption/desorption processes, the double-layer region, and the region of OH adlayer formation, respectively. Modified and reproduced with permission from ref (66), 2007, AAAS.

Rigorous thin-film studies carried out in UHV have become the standard by which fundamental phenomena are unraveled at the atomic level and are applied liberally in the study of ligand effects in electrocatalysis.^62^ To understand the how the ligand effect advances activity, Stamenkovic et al.^40^ applied an UHV surface science approach to the study of Pt and Pt–M surfaces. Thermal annealing expedites Pt surface segregation toward an outer “Pt-skin” structure on Pt_3_Co and Pt_3_Ni films, an observation which correlates well with the seminal theoretical work by Ruban et al.,^67^ who predicted the segregation of host and foreign metals in bimetallic systems from thermodynamic first principles. The formation of the “Pt-skin” was explored by LEIS and AES (Figure 6a,b), which provide complementary information. The LEIS results show the composition of the topmost atomic layer, and AES data are used to analyze the surface composition of a few atomic layers beneath the topmost surface. In LEIS, only the Pt peak can be observed, whereas both Pt and Ni signals are displayed in AES results.^68^ Stamenkovic et al.^66^ later demonstrated that introducing a single atomic layer of Ni beneath the Pt-skin results in a 10-fold increase in ORR activity on model surfaces, where the ligand effect directly induces electronic structure modification and subsequent d-band downshift to reduce the number of unfilled antibonding states. With a decrease in OH adsorption energy and hence OH coverage, the availability of surface sites for O–O bond cleavage increases, illustrated by the current trace between 0.6 and 0.8 V vs RHE in Figure 6c and 100 mV positive shift in half-wave potential in Figure 6d.^66^ The benefits of a single atomic layer beneath the Pt(111) surface provide strong evidence for the influence of electronic structure modification on catalytic activity. The polarization profiles for both Pt(111) and Pt_3_Ni(111) exhibit similar features, suggesting that Pt_3_Ni alloys have the same ORR pathway as pure Pt (Figure 6d).^40,66^

**Figure 7:**
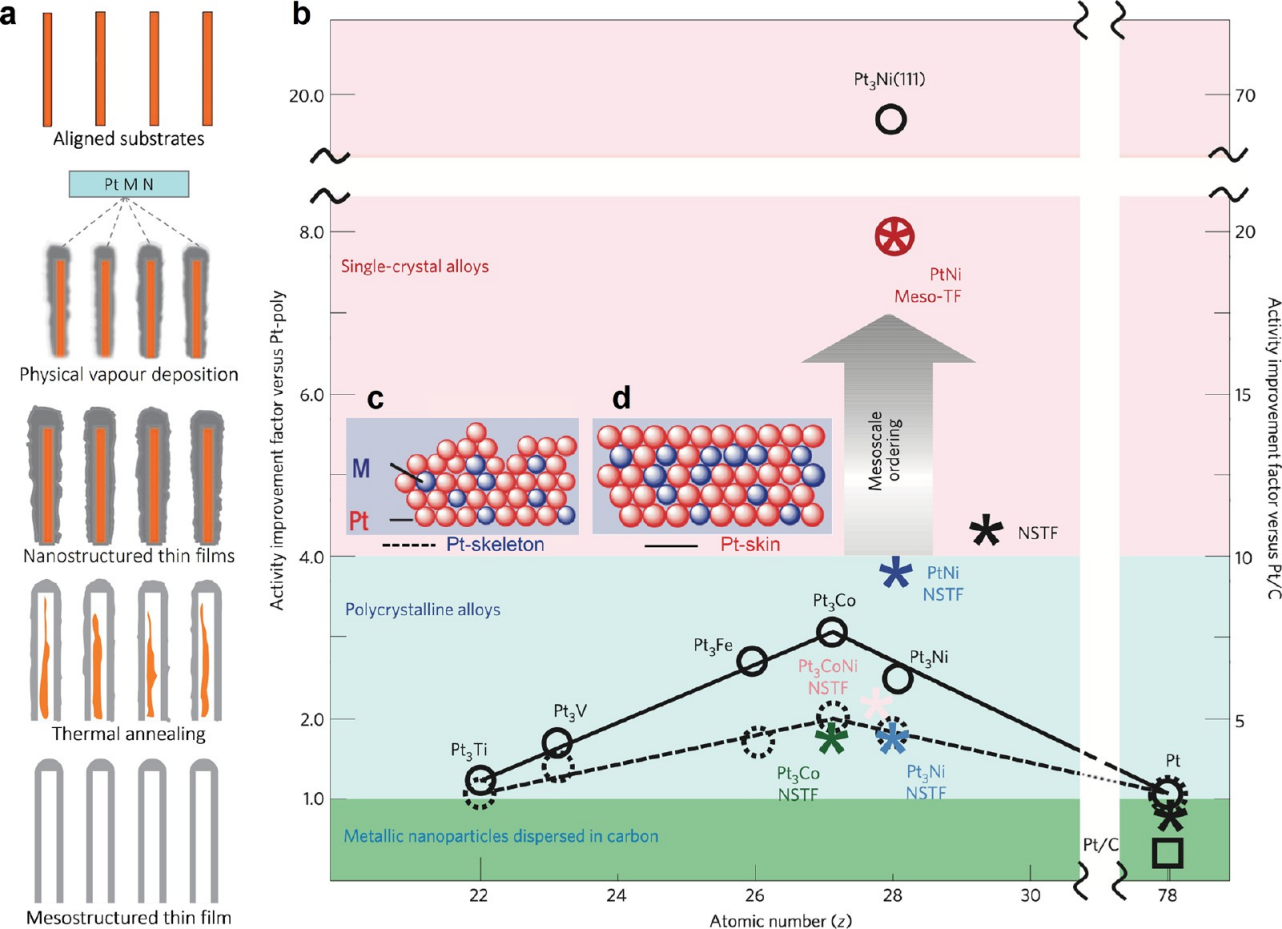
(a) Schematic illustrations of the preparation procedure of the mesostructured thin film. (b) Activity map for the ORR obtained for different classes of Pt-based materials. Improvement factors are given on the basis of activities compared with the values for polycrystalline Pt and the state-of-the-art Pt/C catalyst established by RDE measurements in 0.1 M HClO_4_ at 0.95 V vs RHE. Modified and reproduced with permission from ref (69), 2012, Springer. Schematic models showing that (c) Pt-skeleton and (d) Pt-skin are stable surface formations in the electrochemical environment. Modified and reproduced with permission from ref (19), 2007, Springer.

Mesostructured thin films serve as a bridge between the extended single-crystal surfaces with outstanding catalytic activity and the nanomaterials with significantly larger specific surface area required for practical applications.^69^ The preparation procedure of Meso-TF catalyst is schematically illustrated in Figure 7a, where nanostructured thin films (NSTFs) are first obtained by physical vapor deposition followed by substrate evaporation and thermal annealing to induce formation of the Pt-skin.^69^ Meso-TF has demonstrated exceptional ORR activity that is 20 times higher than that of conventional nanoparticulate ORR catalysts. The significant activity enhancement can be attributed to a near-surface structure similar to that of the ideal extended Pt_3_Ni(111)-skin surface (Figure 7b).^69^ It is, however, crucial to distinguish between Pt-skin and Pt-skeleton structures due to the significant differences in the electrochemical properties of each surface. Though each may be terminated by Pt atoms at the surface, the morphology and concentration profile of the host and foreign metal is distinct in each case (Figure 7c,d). The Pt-skin exhibits a perfectly uniform surface, contrasting the Pt-skeleton which is corrugated and has a concentration profile resembling that of a bulk alloy.^19^ The Pt-skeleton is obtained after immersion of as-sputtered Pt–M alloy in HClO_4_ electrolyte and is terminated by Pt in the same manner as the skeleton structure, as the 3d element dissolves. The surface ordering is decisive in ensuring the stability of the surface for long-term operation in acidic electrolytes.

The electrochemically active surface area (ECSA) of Pt-based catalysts can be determined by hydrogen underpotential deposition (H_upd_) and CO stripping experiments through integration of charge beneath the cyclic voltammogram (CV).^71^ To confirm the existence of the Pt-skin surface, Van der Vliet et al.^70^ assessed the ratio of integrated charge between H_upd_ and CO stripping, *Q*_CO_/2*Q*_H_. Experimental results indicate Pt-skin surfaces have a ratio of CO to hydrogen adsorption greater than unity when compared with single or polycrystalline Pt surfaces (Table 1).^70^ The deviation of the integrated charge ratio from unity is a consequence of suppressed hydrogen surface coverage, as the electronic structure is altered by the ligand effect of atomic arrangement beneath the Pt-skin. This unique characteristic of the Pt-skin surface affords the use of integrated charge ratios to assess the presence of Pt-skin structures on nanoparticles and thin films.^70^

**Table 1:** Integrated Charges and Calculated Surface Areas (ECSAs) for H_upd_ (*Q*_H_) and CO Stripping (*Q*_CO_) Obtained from Cyclic Voltammetry of Pt/C, Acid-Treated PtNi/C, and Annealed PtNi/C Catalysts^a^

catalyst	*Q*_H_ (μC)	*Q*_CO_ (μC)	*Q*_CO_/2*Q*_H_
Pt(111)	152	315	1.04
Pt_3_Ni(111)	98	304	1.55
Pt/C	279	545	0.98
PtNi/C	292	615	1.05
PtNi skin/C	210	595	1.42

a The ratio between the integrated charges for H_upd_ and CO stripping demonstrates the discrepancy in ECSAs and the underestimation of the real surface area if H_upd_ is used in the case of Pt-skin surfaces. Modified and reproduced with permission from reference (70), 2012, Wiley.

**Figure 8:**
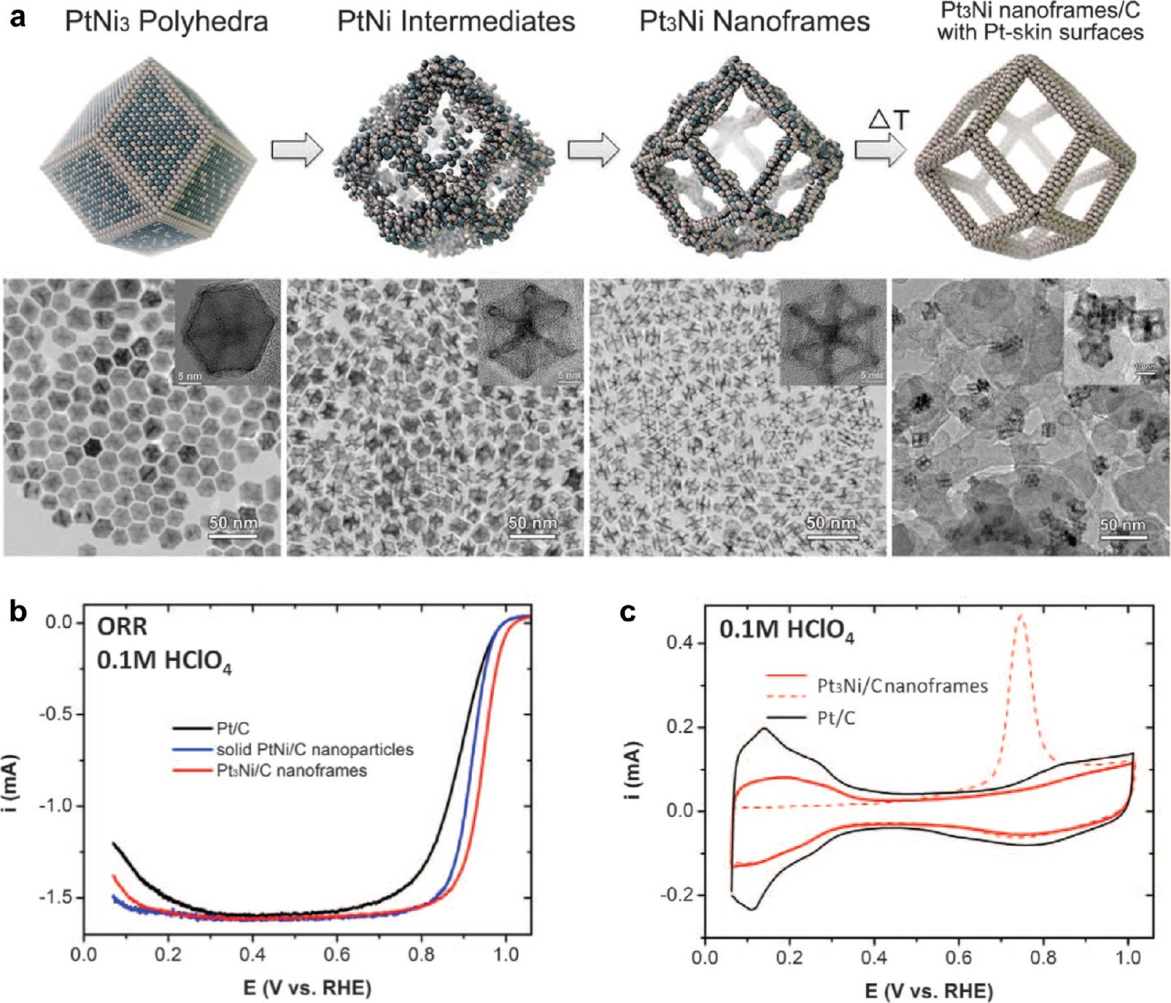
(a) Schematic illustrations and corresponding TEM images of the samples obtained at four representative stages during the evolution process from polyhedra to nanoframes. Electrochemical properties of Pt_3_Ni nanoframes. (b) ORR polarization curve. (c) Cyclic voltammograms of Pt/C and Pt_3_Ni/C nanoframes signifying the difference in surface coverage by H_upd_ and OH_ad_. Modified and reproduced with permission from ref (72), 2014, AAAS.

Given the extraordinary activity of Pt-skin surfaces, Chen et al.^72^ synthesized a Pt_3_Ni nanoframe catalyst (Figure 8a) based on this concept. The ordered hollow architecture minimizes buried nonfunctional material and provides three-dimensional (3D) surface site accessibility, while the Pt-skin termination reduces OH coverage to maximize activity. By combining the exceptionally high geometric surface area of the nanoframe with lower OH* coverage induced by the Pt-skin, a 36-fold improvement in mass activity is observed as shown in Figure 8b, the ORR polarization curve. The CO-stripping data in Figure 8c provide clear evidence for the presence of the Pt-skin structure as *Q*_CO_/2*Q*_H_ deviates significantly from unity, measured at 1.52.^72^ Similar observations with regard to the influence of subsurface alloying have been made by other groups. Stephens and co-workers examined Pt–Cu near-surface alloys through variation of the subsurface copper concentration.^73^ An optimal copper concentration of 0.5 monolayer (ML) beneath the surface lies at the apex of a volcano-type plot and yields an 8-fold activity increase over Pt(111) at 0.9 V vs RHE. In these examples, a single transition-metal layer or less beneath the Pt skin is responsible for a tremendous increase in activity, providing the best evidence of the ligand effect improving reaction kinetics for the ORR across a range of length scales, to our knowledge.

Kitchin et al.^74^ developed a DFT model comparing the ligand effect of early to late transition metals, supporting the findings of Stephens and colleagues that the ligand effect may be sufficient to explain the activity improvements of Pt–M alloys. Their results showed that Pt–M coupling widens and shifts the d-band, resulting in altered O_2_ and H_2_ adsorption properties at the surface. Replicating activity both observed and calculated on model surfaces in practical systems is an ongoing challenge, but by tuning the surface electronic structure, impressive performances have been recorded, emphasizing the importance of the ligand effect in extending the unprecedented activity of model surfaces to real devices.^13,14,40,64−66^

### 3.2 Strain Effects

**Figure 9:**
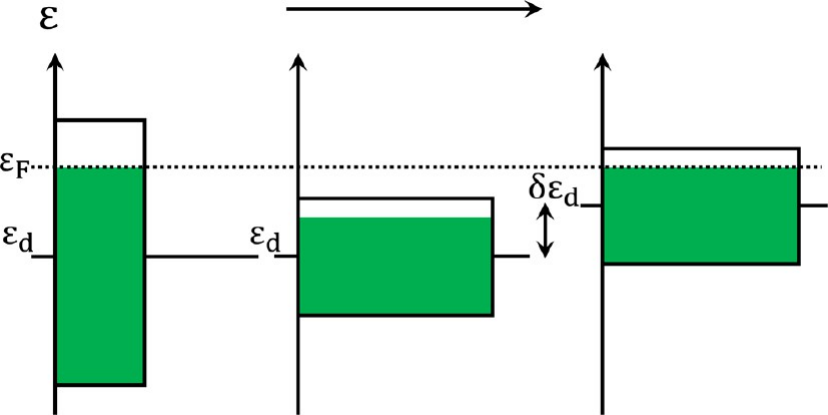
Schematic illustration of the coupling between bandwidth and d-band center for a band with a fixed number of d-electrons. When the bandwidth is decreasing, the only way of maintaining the number of d-electrons is to shift up the center of the band. Reproduced with permission from ref (65), 2007, Elsevier.

The distribution of electrons in the d-band is directly influenced not only by subsurface dissimilar elements but by lattice strain and coordination number as well.^9,64,75^ Each atom in a metal lattice has a distinct electric character depending on its environment, giving every atom an influence over others within the coordination sphere; this is the basis of the ligand effect. The exact nature and magnitude of this interaction depends on the distance between atoms and the lattice parameter such that changes to lattice strain will affect the d-band of a metal.^9,76,77^ For example, a larger lattice parameter, or greater lattice spacing, lessens the influence of neighboring atoms and condenses the energy distribution of the d-band, as shown from the left to the right of Figure 9.^65^ As the number of electron levels that are filled beneath the Fermi level will not change, the center of the d-band shifts up to stabilize the number of filled states. Adsorbate binding energy to the strained surface is altered in much the same manner as ligand effects: tensile strain shifts the d-band closer to the Fermi level, which will leave the high-energy antibonding orbitals unfilled and increase adsorbate binding strength. The opposite is also the case, meaning that compressive strain will lower the d-band and decrease bonding strength,^65^ which in the case of the Pt ORR systems is the desired effect to increase surface site availability.^66^

Experimental evidence exists to support the theoretical connection between strain and activity in the ORR.^9,15,16,18,76−79^ Notably, Strasser et al.^16^ found a distinct d-band shift from above to below the Fermi level on a single-crystal surface in response to compressive strain, which is accomplished by tracking the occupation of bonding and antibonding states of Pt/Cu(111) surfaces. The technique was applied to pure Pt(111) and compared to Cu(111) with 2.6 and 3.5 MLs of Pt which correlate to 2.8% and 3.3% strains, respectively. Through application of XAS and XES, the authors suggest that increasing strain pushes the electrons toward antibonding states, leading to weakened adsorbate binding.^73^ The authors predicted a volcano plot with a maximum activity occurring between 2% and 3% compressive strain, but experimental observations show the maximum activity continues to increase, with a peak occurring at greater than 4% compressive strain. The contribution of the ligand effect was not considered in this work, as the authors suggested strain effects will account for any change in activity, contrasting with the claims by other groups.^80^

**Figure 10:**
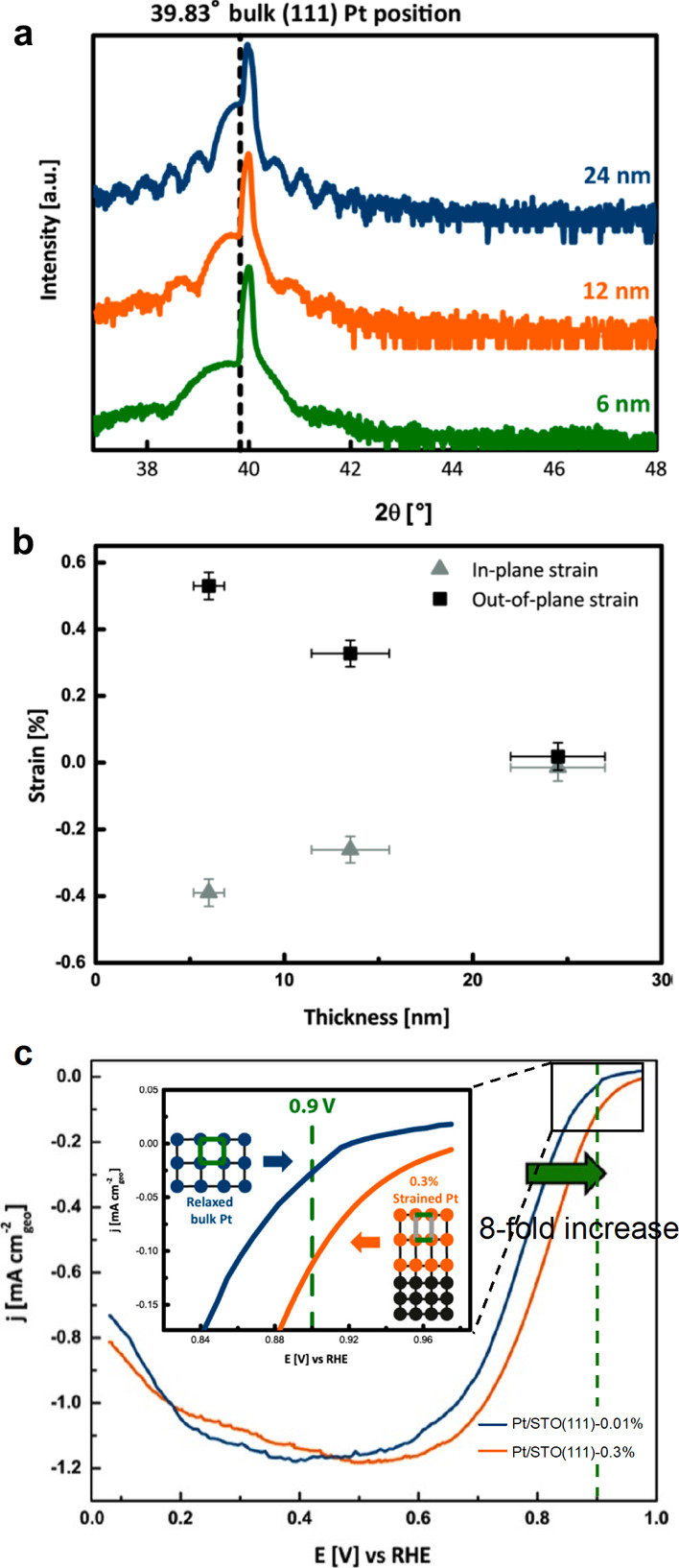
(a) X-ray diffraction patterns of 6, 12, and 24 nm thick Pt films on a SrTiO_3_ substrate. The shift of the (111) Pt peak toward smaller 2θ angles becomes more pronounced with a decrease in film thickness. (b) Both in-plane and out-of-plane strain plotted vs the respective Pt film thickness. (c) Cathodic oxygen reduction reaction polarization curves measured at 10 mV s^–1^. The inset shows the remarkably higher activity of “strained” Pt films compared to that of “relaxed” Pt. Modified and reproduced with permission from ref (81), 2016, American Chemical Society.

Investigations involving core–shell nanoparticles and thin-film studies dominate attempts to study the influence of strain on oxygen electrocatalysis.^15,16,76^ The evidence provided thus far is somewhat questionable, as a significant degree of uncertainty and variation can occur, particularly in the preparation of nanoparticles which are not suitable for fundamental studies of catalytic phenomena. The method of preparation, particle size, and distribution of elements per particle all contribute to considerable uncertainty, which can enhance or hinder the ORR performance, especially when studying electronic effects at the catalyst surface. To mitigate the electronic influence on the topmost Pt layer, Temmel et al.^81^ introduced the pulsed laser deposition (PLD) technique to fabricate Pt films of varying thicknesses (6, 12, and 24 nm) on an insulating strontium titanate (SrTiO_3_, STO) single-crystal (111) substrate (Figure 10). This approach removes potential side effects caused by electronic interferences prevalent in electrochemical studies with nanoparticle catalysts as the subject. Evaluation of in-plane lattice strain (ε_*XX*_) is crucial, since ε_*XX*_ can modify the d-band center of Pt surfaces. In-plane lattice strain can be calculated by eq 2(81)
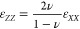
2where ν is the Poisson ratio^82^ and ε_*ZZ*_ is the out-of-plane tensile strain, which can be directly evaluated from the epitaxially grown Pt film from peak position analysis in XRD measurements. Figure 10a reveals the negative peak shift for the 6 and 12 nm Pt films, demonstrating that a shift in ε_*ZZ*_ is induced due to the difference in lattice parameters of the STO(111) substrate and the Pt overlayer. The estimated strains for each sample are displayed in Figure 10b, revealing that ε_*XX*_ values of the 6 and 12 nm Pt films are negative, −0.4% and −0.3%, respectively, suggesting that compressive strain is impressed on the Pt surface. At 24 nm thickness, Pt films show almost zero strain for both in-plane (+0.01%) and out-of-plane (−0.01%), demonstrating that the surface becomes independent of the substrate above a certain thickness. The ORR activities of 12 nm (strained) and 24 nm (relaxed) Pt films were compared, displaying 8-fold activity enhancement in the strained sample (12 nm) at 0.9 V vs RHE. The compressive strain of the Pt surface led to substantial activity improvement (Figure 10c), though neither EELS nor XPS studies were conducted to assess the d-band state. It is worth highlighting that XRD reveals only the average strain over the whole film thickness and that in-plane strain is not directly translatable to absolute surface strain. Although only a qualitative trend in surface strain can be assessed in this study, this research provided the first experimental method for direct evaluation of surface strain via XRD measurement.

**Figure 11:**
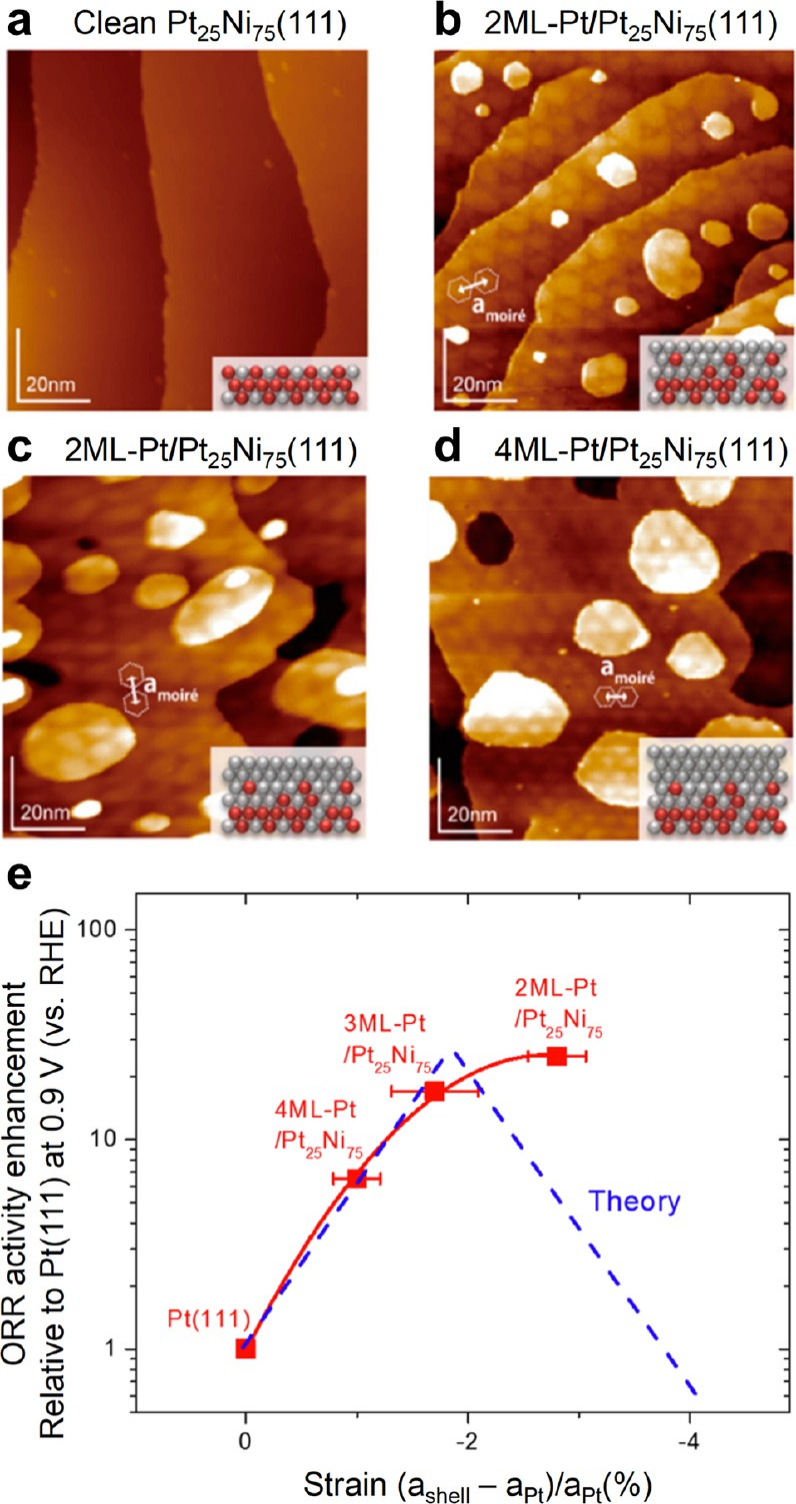
UHV–STM images for (a) a clean Pt_25_Ni_75_(111) surface, (b) a 2 ML-Pt/Pt_25_Ni_75_(111) surface, (c) a 3 ML-Pt/Pt_25_Ni_75_(111) surface, and (d) a 4 ML-Pt/Pt_25_Ni_75_(111) surface. Insets are atom models of the corresponding surfaces. Degrees of ORR enhancement at 0.9 V for the *n*ML-Pt/Pt_25_Ni_75_(111), relative to Pt(111), as a function of surface strain. Reproduced with permission from ref (83), 2016, American Chemical Society.

In a novel approach, Asano et al.^83^ investigated the effect of strain on ORR activity by preparing well-defined bimetallic Pt–Ni alloy surfaces through molecular beam epitaxy (MBE). Epitaxially grown Pt MLs on Pt_25_Ni_75_ (111) surfaces exhibit moiré patterns in scanning tunneling microscopy (STM) images (Figure 11a–d), generally originating from the lattice mismatch between the first and second atomic layers.^83^ The highly ordered patterns imply a uniform lateral strain on the topmost Pt layer in long-range order, induced by the lattice mismatch. The lattice strain can be calculated by eqs 3 and 4 based on the periodicities of the patterns^83^
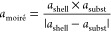
3
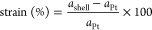
4where *a*_moiré_ is the center-to-center distance of moiré patterns measured from the STM images. *a*_shell_, *a*_subst_, and *a*_Pt_ are lattice constants of the topmost Pt layer, PtNi_3_ substrate, and bulk crystalline Pt, respectively. Figure 11e displays the ORR activity enhancements of the *n*ML-Pt/Pt_25_Ni_75_(111) and clean Pt surface as a function of the strain on the topmost Pt layer.^83^ The ORR activities of 3 and 4 ML-Pt/Pt_25_Ni_75_(111) match well with theoretical predictions. Interestingly, although a decline in catalytic activity is predicted when the strain crosses the optimal value, the 2 ML-Pt/Pt_25_Ni_75_(111) sample shows much higher catalytic activity than expected. The observation highlights the strong efficacy of ligand effects introduced by subsurface Ni beneath 2 ML-Pt, outweighing the detrimental effect of inducing compressive strain beyond the theoretically optimal value on ORR activity.

**Figure 12:**
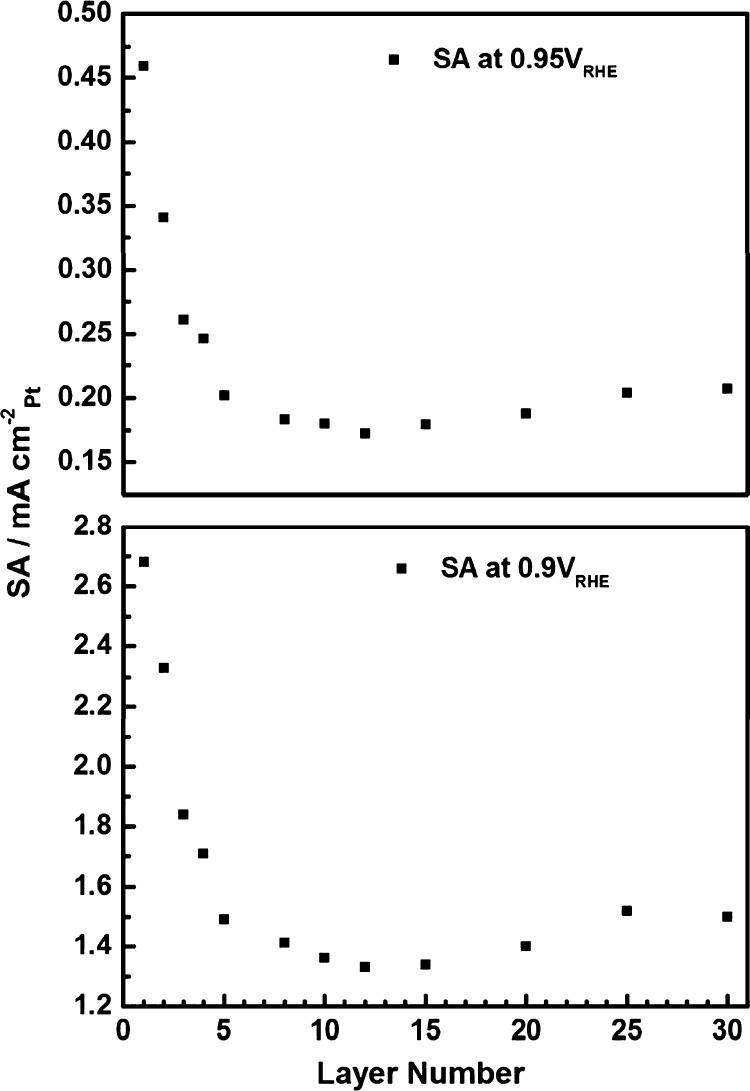
Comparison of the specific ORR activity SA at 0.9 and 0.95 V vs RHE as a function of the number of nominal Pt layers deposited onto the Au film electrodes. Reprinted with permission from ref (18), 2016, American Chemical Society.

Au has conflicting attributes as an alloy with Pt, as its electronic structure is believed to have a positive ligand effect, while the larger atomic radius and associated increase in lattice spacing is expected to inflict undesired tensile strain on the Pt-skin surface.^84,85^ Deng et al.^18^ investigated the continuous change in activity as Pt MLs were deposited onto a polycrystalline Au film, finding that at 1–3 MLs, the ligand effect of the Au film induces a positive shift in activity (Figure 12). From this point, activity decreases to a minimum, eventually approaching that of polycrystalline Pt. The short-range nature of the interaction between the Pt surface and the Au beneath supports the positive net effect of Au on surface electronic structure and relegates strain to a secondary phenomenon, which in the specific case of Au and Pt, competes with the ligand effect to determine d-band position and the strength of adsorbate–surface interactions. A second case of competing effects was investigated by Johansson et al.^86^, where theoretical predictions suggest a Pt-skinned Pt_3_Sc will display inferior catalytic activity for the ORR, compared to a polycrystalline Pt surface caused by tensile strain exerted on the surface by much larger Sc atoms. Surprisingly, activity increased, providing further evidence that in cases of competing effects, electronic structure modifications are more strongly affected by interactions with adjacent dissimilar elements than lattice spacing.

### 3.3 Ensemble Effects

The ensemble effect in electrocatalysis can be described as a particular arrangement of surface atoms which favors a certain reaction and may be bifurcated into cases with dissimilar elements and dissimilar arrangements of the same element in small groups or ensembles.^12,87^ In either case, the dissimilar factions play a different mechanistic role: i.e., catalysis of a different reaction step or promotion of different species adsorption. Ensemble effects are known to be significant in the oxidation and reduction of small organic molecules, as the adsorbate may interact with the surface at multiple points or in some cases coadsorption through a Langmuir–Hinshelwood type mechanism can be required for the reaction to progress.^12,21,88−91^ For the case of O* and H* binding energies, Li et al.^92^ found that ensemble effects contribute to significant changes in adsorption strength where elements with strong and weak affinity for the adsorbate are paired. The effect is less pronounced where binding energies of the elements are similar, leading ligand and strain effects to be more important. Deng et al.^18^ proposed ligand and ensemble effects act in concert for the ORR on PtAu, suggesting surface Au decreases the oxygen binding energy of the surface. However, Au on Pt surfaces tends to deposit initially at step/edge sites, and at coverages of 0.2 ML, a negligible effect on activity is observed.^24^ The study of ensemble effects is clouded by investigations based on imperfect nanoparticles with limited surface uniformity.^93,94^

As the field further develops PGM catalysts for the ORR, it is worth noting that there is experimental evidence in support of both the ligand and strain effects in bimetallic catalysts. DFT calculations and d-band theory can account for both effects to a varying extent, but further experiments are required to truly decouple the two effects and fully describe the strength of each individual effect with respect to the TMs available for these alloyed catalysts.

## 4 Experimental Approaches

### 4.1 *Ex Situ* Techniques for Material Characterization

**Figure 13:**
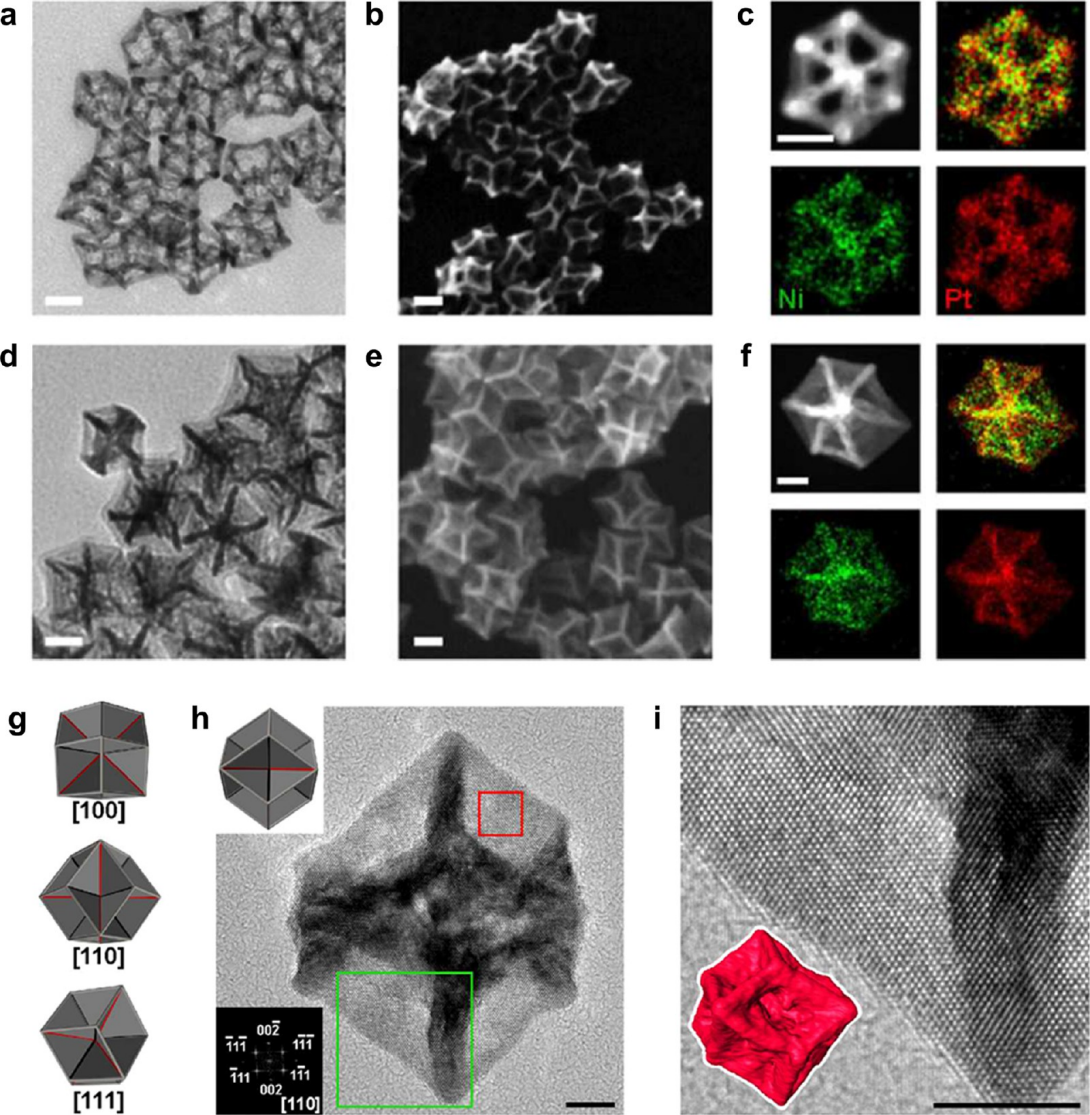
TEM and SEM images of (a, b) a hollow nanoframe and (d, e) a excavated nanoframe (E-NF). STEM-HAADF image and STEM-EDS mapping of (c) a hollow nanoframe and (f) a excavated nanoframe oriented in the ⟨111⟩ direction. (g) Model of E-NF shown in three orientations. (h) HRTEM image of E-NF oriented in the ⟨110⟩ direction. The top left inset is the corresponding model of ENF in the identical orientation. The bottom left inset is the FFT of the image in the red box. (i) Magnified HRTEM image of thin sheet in E-NF from green box in (h), with high-tilt angle STEM tomography rendering of E-NF in the inset. Scale bars represent 10 and 5 nm for (a–f) and (h, i), respectively. Reprinted with permission from ref (99), 2017, American Chemical Society.

Bimetallic catalysis has created opportunities to improve activity for multiple reactions through tuning of ligand, strain, and ensemble effects.^15,16,95^ Concurrently, however, it has generated questions and uncertainty around structure, degradation, and reaction mechanisms and how such processes can be experimentally evaluated and quantified. Typically, electrochemical characterization will be supported with a variety of *ex situ* techniques including TEM, STM, HAADF-STEM, and XRD to provide information on the structure of the nanoparticle catalysts from average particle size to the presence of different crystalline phases.^23,27^ Pertinent in bimetallic catalysis, however, is the use of STEM-EDS, which has been used to great effect for the determination of elemental composition, with nanoframe and intermetallic structures being two key examples which can be identified using this approach (Figure 13).^96−99^

*Ex situ* techniques provide extensive information on an as-synthesized material and the subsequent changes undergone by a catalyst during operation. To uncover specific conditions under which changes occur, *operando* methods are necessary where for bimetallic catalysis dissolution can be correlated to reaction conditions or applied potential. In this section, we will focus primarily on novel approaches with relevance to bi- and multimetallic catalysis with transition metals, as several comprehensive reviews of *in situ* and *operando* techniques can be found in the literature.^100,101^ Early-stage development studies of degradation for Pt–M catalysts are critical for identification of promising candidates for electrocatalysis which offer superior durability over conventional single metal catalysts. To achieve the 35000 h target for heavy duty fuel cell trucks, knowledge of the relationship between applied potential and catalyst degradation is paramount to engineering new solutions with application in real systems.^102^

### 4.2 Aqueous *Operando* Methods

In recent years, several groups have effectively combined inductively coupled plasma mass spectrometry (ICP-MS) with electrochemical analysis techniques.^103−105^ Combining a flow cell with ICP-MS affords insight into catalyst dissolution in real time, as the concentration of the active metal in solution can be determined as a function of time and applied potential. Introduced by Klemm et al. in 2011, the scanning flow cell inductively coupled plasma mass spectrometry (SFC-ICP-MS) technique has developed into a key tool for understanding relationships between structure and stability at the fundamental level. In their seminal work, the authors were able to prove the feasibility of the technique with a scanning flow cell to measure the dissolution of copper in HCl solution,^106^ before applying the same approach to Pt dissolution in acidic media. Through this approach, the authors showed, for the first time, higher levels of Pt dissolution during the cathodic rather than the anodic scan, indicating the important role of Pt oxidation and reduction and what would later be termed the “place exchange mechanism”.^107^

**Figure 14:**
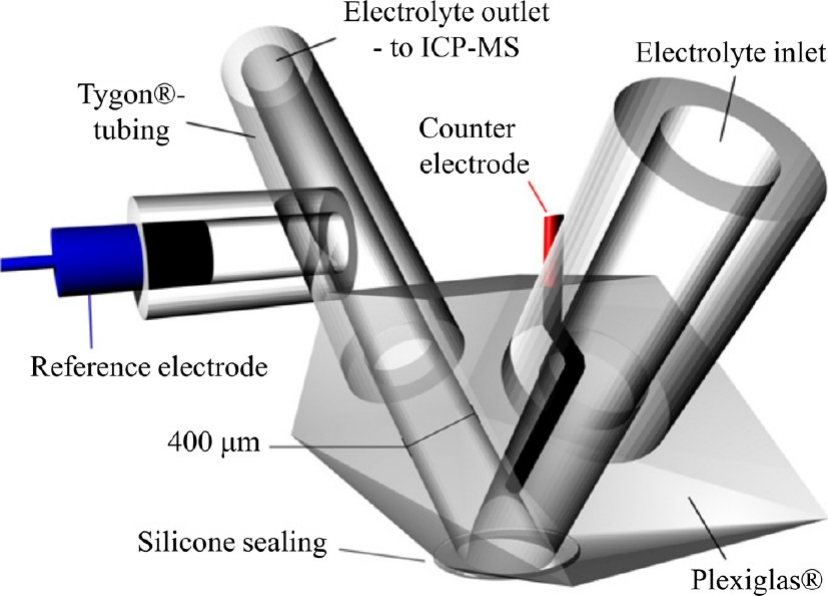
Scanning flow cell (SFC) configuration for coupling electrochemical cells with in-line ICP-MS providing potential and time-resolved dissolution measurements. Modified and reprinted with permission from ref (106), 2011, Elsevier.

As Weiss commented in his 2012 article for ACS Nano, “new tools lead to new science”, an apt statement for the addition of in-line elemental analysis to the field of electrochemistry and associated stability studies.^108^Figure 14 shows a schematic diagram of the setup used by Mayrhofer’s group, in which the electrolyte flows through a sealed Plexiglas container, with the outlet connected to the ICP-MS.^106^ Multiple working electrodes can be arrayed on a surface such that the Plexiglas container may be moved to seat on each working electrode, allowing multiple cells to be tested in succession. This approach is particularly beneficial in cases where a high degree of uncertainty in ink recipe or performance might be expected.

**Figure 15:**
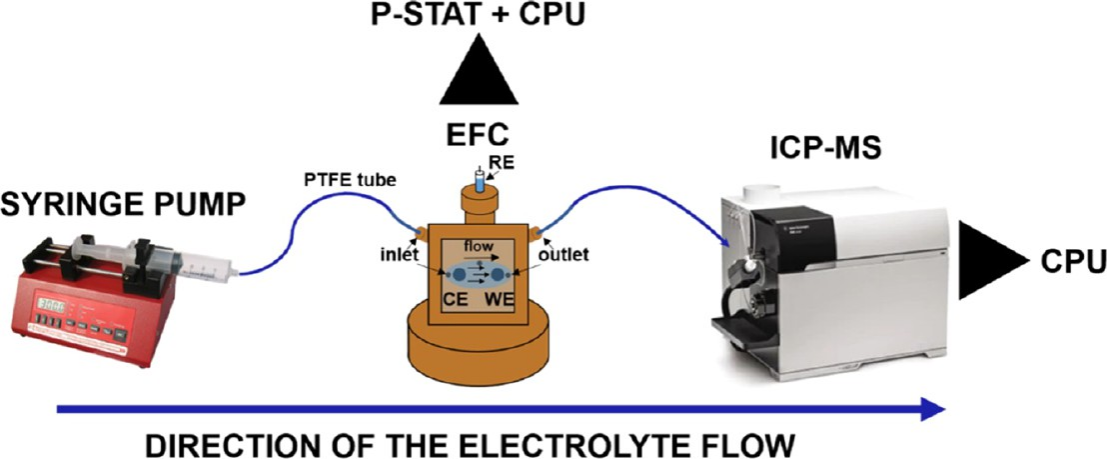
Electrochemical flow cell (EFC) configuration for coupling electrochemical cells with in-line ICP-MS providing potential and time-resolved dissolution measurements. Reprinted with permission from ref (109), 2022, American Chemical Society.

Building on this early work, Jovanovic et al. applied in-line ICP-MS to measure the amount of metal dissolved during potential cycling of commercial Pt/C and prepared core–shell PtCu/C catalysts for the ORR.^104^ With a slightly different approach, the authors employed a setup in which a single cell is connected directly to the ICP-MS (Figure 15).^104^ For cycles with the same upper potential applied, the results showed an excellent reproducibility of ±3%. This work, however, served to demonstrate the challenges associated with Pt–M alloy durability, as PtCu/C had higher levels of dissolution compared to commercial Pt/C during potential cycling, suggesting the introduction of a transition metal does not always improve the durability of the catalysts.

**Figure 16:**
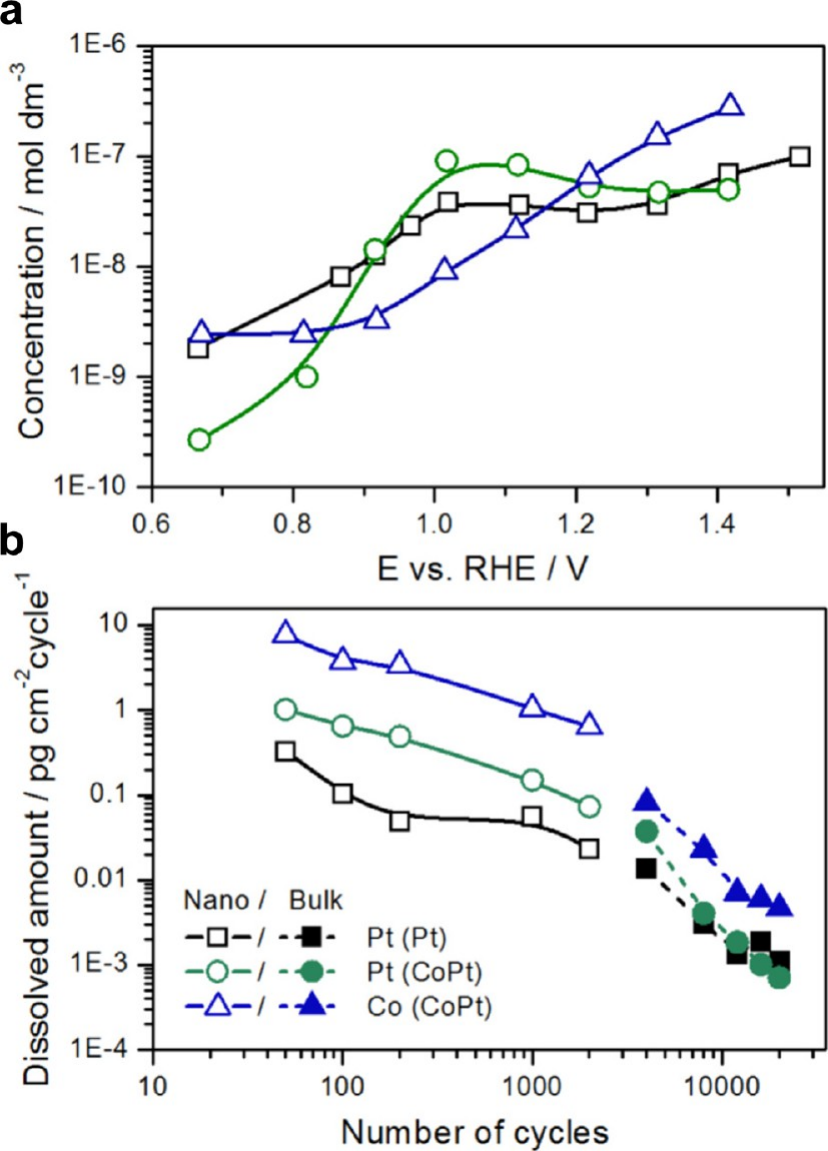
(a) Dependence of equilibrium concentration of Co and Pt in contact with Pt/C and Co–Pt/C electrodes. (b) Variation of Pt and Co dissolution with potential cycle. Reprinted with permission from ref (110), 2016, Elsevier.

Cherevko et al. applied the electrochemical flow cell technique to bimetallic PtCo in the first study to assess the dissolution of different metallic elements independently, shown graphically in Figure 16, where the concentration of both Pt and Co are plotted as a function of (a) potential and (b) cycle number.^110^ Ahluwalia et al. assessed PtCo/C to examine the dependence of dissolution on Pt–O_*x*_ formation,^111^ with results indicating that Co dissolution occurs with both potential-dependent and potential-independent mechanisms, regardless of the upper potential limit across a wide potential window. This contrasts with well-known Pt dissolution mechanisms involving Pt-oxide formation and reduction near 0.8 V vs RHE.^112^ With a combination of standard and novel techniques, the Gaberšček group measured different responses to electrochemical activation protocols for Pt–M alloys.^113^ With specific reference to ICP-MS, the authors were able to show that the dealloying behavior for Co and Cu with Pt is comparable but subsequent Pt–M interactions are starkly different due to stabilization of copper through thermodynamic deposition on the Pt surface, a process which is not observed for cobalt. Stabilization allows the copper to remain adjacent to the Pt surface for a longer period, manifesting as overpotential deposition in cyclic voltammograms as it is stripped and redeposited. Bogar et al. used ICP-MS to study the dissolution of nickel from Pt–M alloys to find that dissolution of the less noble metal is the primary cause of degradation when a lower upper potential limit (UPL) is applied. With higher UPLs, there exists a transition between dissolution, coalescence, and Ostwald ripening as the major contributor to degradation as a function of cycle number.^114^

In a subsequent study, Gatalo et al. used ICP-MS to examine the effect of *ex situ* chemical activation protocols on Pt-alloy catalysts.^109^ Leaching of less noble metals from multimetallic electrocatalysts is to a certain degree unavoidable; however, it is critical to minimize the extent to which this occurs *in situ*, as transition-metal ions may go on to accelerate system degradation through Fenton reactions.^115^ However, there is evidence that it may be possible to reduce or eliminate dissolution to below the limit of detection of some modern mass spectrometers. With an Au@Pt core–shell system, Ledendecker et al. were able to show elimination of gold dissolution in potential cycling experiments.^116^

In 2016, Lopes et al. developed the RDE-ICP-MS method for use of the ICP-MS with the rotating disk electrode technique. The novel aspect of coupling mass spectrometry to a rotating disk electrode lies in the link between applied potential and real-time dissolution characteristics under well-defined diffusion/kinetic control.^103^ In this work, the authors correlated the dissolution of surface atoms from single-crystal electrodes to kinetic rates of electrochemical reactions. Using the same method, the authors later proposed two distinct mechanisms for Pt dissolution during oxide formation and reduction as described in earlier sections. During the anodic scan, electrochemical dissolution is dominant, whereas in the cathodic scan, both electrochemical and chemical dissolution are present:^117^
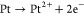
5
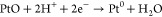
6
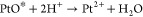
7

**Figure 17:**
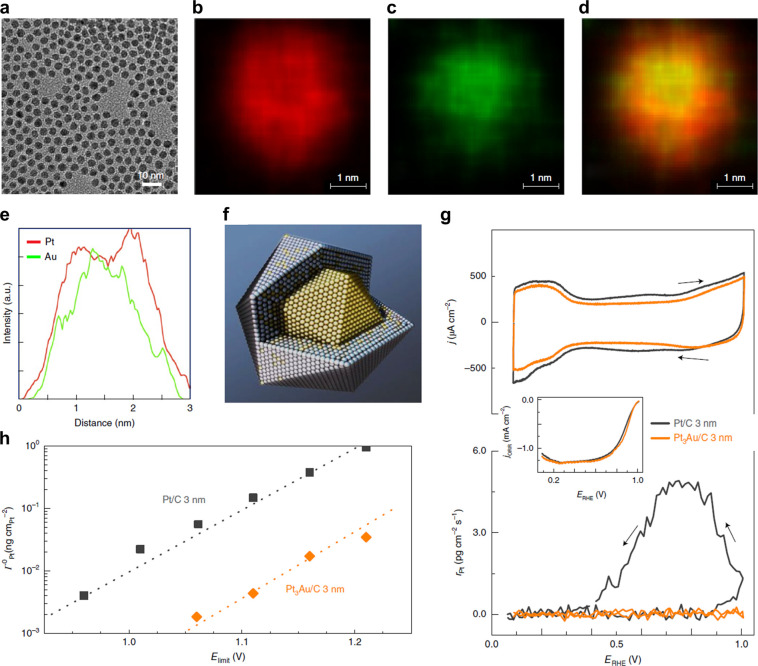
Combining *ex situ* and *in situ* techniques for evaluating Pt_3_Au NPs. (a) TEM confirming a uniform size distribution (3 nm). (b–d) HR-STEM along with EDS mapping of Pt, Au, and overlaid Pt/Au images (d). (e) EDS composition line scan revealing an Au-rich core with a Pt shell. (f) Illustration of Pt_3_Au core–shell NP with a distinct compositional gradient. (g) Cyclic voltammograms and corresponding Pt dissolution profile for Pt/C and Pt_3_Au/C in 0.1 mol L^–1^ HClO_4_. Inset: ORR polarization curves. (h) Comparison between the intrinsic dissolution of Pt for different positive potential limits (up to 1.2 V vs RHE), revealing an improvement in Pt stability. au denotes arbitrary units. Reprinted with permission from ref (24), 2020, Springer.

It was not until later that this method was extended to bimetallic catalysts by Lopes et al., in a comprehensive study on PtAu catalysts.^24^ In this work, the authors leveraged the ICP-MS technique in combination with the rotating disk electrode setup to assess the impact of both surface gold atoms and a gold underlayer on the stability of Pt electrocatalysts with varied potential limits. Evidence was presented that the Pt surface is likely to form Pt(111) when supported by subsurface Au while surface Au may act to protect lower coordinated sites prone to dissolution through preferential adsorption. The intrinsic dissolution of the electrocatalyst is shown as a function of upper potential limit for 3 nm nanoparticles of Pt/C and PtAu/C, indicating stability benefits through a reduction of dissolution over a wide range of potentials (Figure 17). Using a combination of *in situ* and *ex situ* characterization techniques, a comprehensive view of catalyst structure, stability, and performance are presented revealing the origin of structure–stability relationships at the atomic level.

Surface modifiers and ionic liquids present an interesting new avenue of research in electrocatalysis.^118^ Several groups have explored the use of organic molecules to decorate the Pt surface and tune specific interactions.^119−123^ Cherevko et al. applied the ICP-MS with scanning flow cell technique to trimetallic PtNiMo/C modified with ionic liquids.^118,124^ Previous work has shown positive effects of ionic liquid modification on single-element Pt catalysts, but the effect on multimetallic catalysts is less clear.^119,125,126^ The dissolution profiles of each of the metals were studied by SFC-ICP-MS. In this case, the ionic liquid promotes leaching of the Mo from the catalyst surface, eliminating the stabilizing effect on nickel. Further work in this area is necessary to understand the interactions of each element incorporated in the electrocatalyst with ionic liquids and surface modifiers.

Recently, the Mayrhofer and Cherevko group applied the in-line ICPMS technique to a gas diffusion electrode (GDE) half-cell.^127^ Using this approach, it is possible to probe the transport of Pt through Nafion membranes, leading to observations that dissolution increases with reductions in loading and that dissolution is lower in GDEs than in aqueous electrolytes, possibly due to mass transport effects influencing dissolution and redeposition within catalyst layers. Further work in this area is needed to understand how Pt dissolution mechanisms determined in aqueous systems translate to the solid electrolytes used in fuel cells. We anticipate the expansion of this approach to multimetallic catalysis in the near future.

### 4.3 Vibrational Spectroscopy

**Figure 18:**
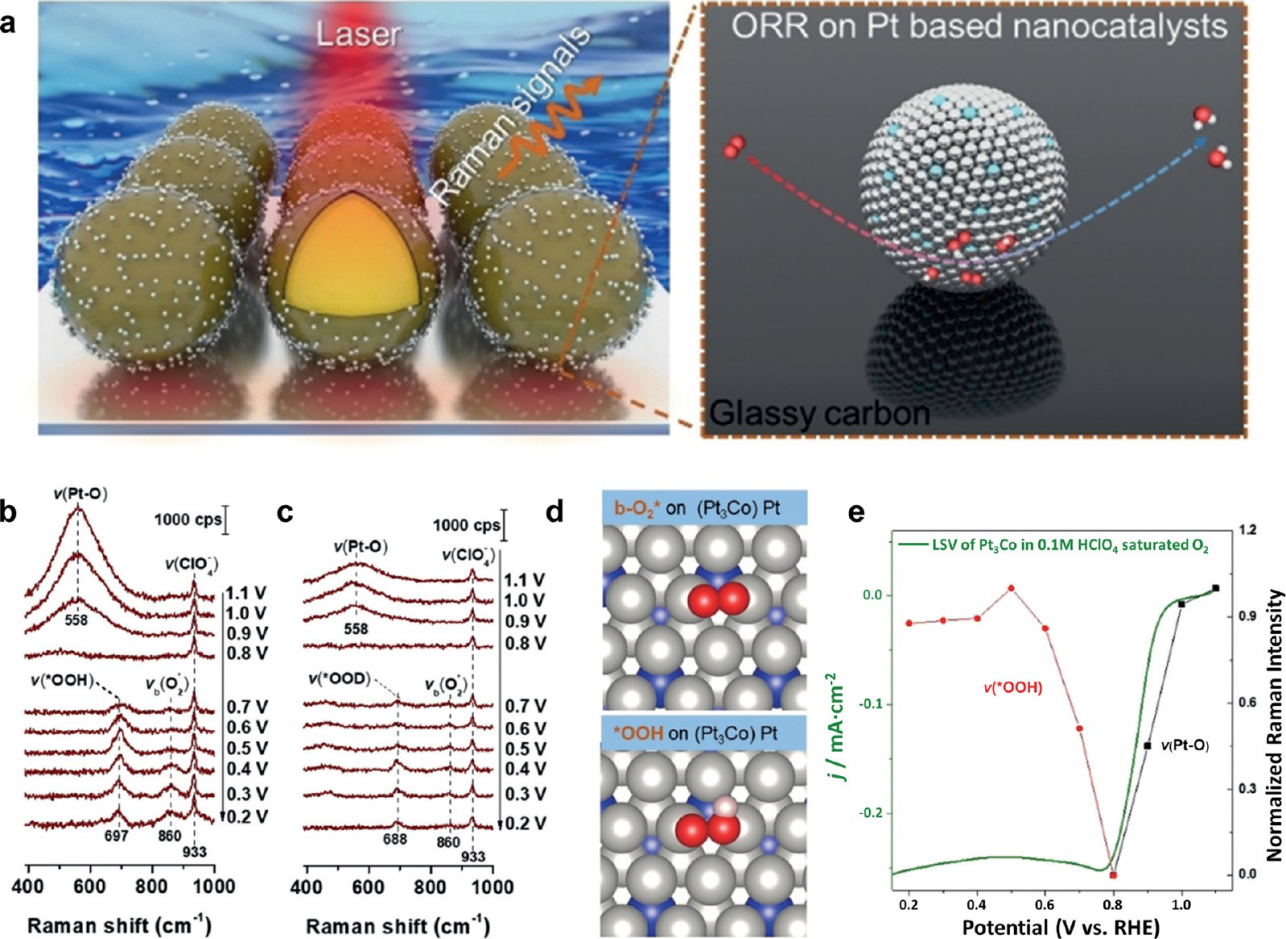
(a) Illustration of EC-SHINERS study of ORR on Pt-based nanocatalysts. *In situ* EC-SHINERS spectra of the ORR on dealloyed Pt_3_Co nanocatalysts in 0.1 M HClO_4_ with O_2_-saturated (b) H_2_O and (c) D_2_O solutions. (d) Adsorption configurations of the ORR intermediate species on the Pt_3_Co surfaces, where the gray, blue, red, and white spheres represent Pt, Co, O, and H, respectively. (e) Normalized Raman intensities of the stretching mode of Pt–O (depicted as black squares) and *OOH (represented as red spheres) as a function of applied potentials. The polarization curve was obtained in 0.1 M HClO_4_ with a scan rate of 1 mV s^–1^. Modified and reprinted with permission from ref (128), 2019, Wiley.

Shell-isolated nanoparticle-enhanced Raman spectroscopy (SHINERS) is an extension of surface-enhanced Raman scattering (SERS)^129^ first developed by Li.^130^ Surface plasmon resonance (SPR) active Au nanoparticles are deposited on the surface of interest, with a controlled silica or alumina shell around each particle to prevent agglomeration and avoid direct contact with the substrate. Each deposited particle serves as an effective tip in a tip-enhanced Raman spectroscopy (TERS) system, introducing a multitude of TERS tips to the substrate’s surface for probing. Consequently, an amplified Raman signal can be collected from entire nanoparticles, leading to significant enhancement of the Raman scattering effect (Figure 18a).^130^ Li et al.^128^ investigated the ORR on Pt_3_Co nanoparticles through electrochemical SHINERS (EC-SHINERS) to gain insight into the effect of transition metals on the reaction mechanism. The presence of hydrogen was confirmed through deuterium isotopic substitution and adsorbed *OH, *OOH, and O_2_* in EC-SHINERS spectra (Figure 18b,c). The *OOH peaks presented slightly lower wavenumbers than on Pt(111), suggesting stronger O–O stretching of adsorbed *OOH due to the weakened adsorption energy onto the Pt_3_Co surface.^131,132^ The O–O stretching of *OOH and *O_2_ with bridge configuration (denoted as b-O_2_*) on surface Pt is graphically illustrated in Figure 18d. Correlation between Raman intensities of the Pt–O and *OOH bands with the ORR performance is displayed in Figure 18e. The disappearance of *OOH from the surface and subsequent formation of Pt–O is correlated to the loss of activity starting at around 0.8 V vs RHE.^128^

**Figure 19:**
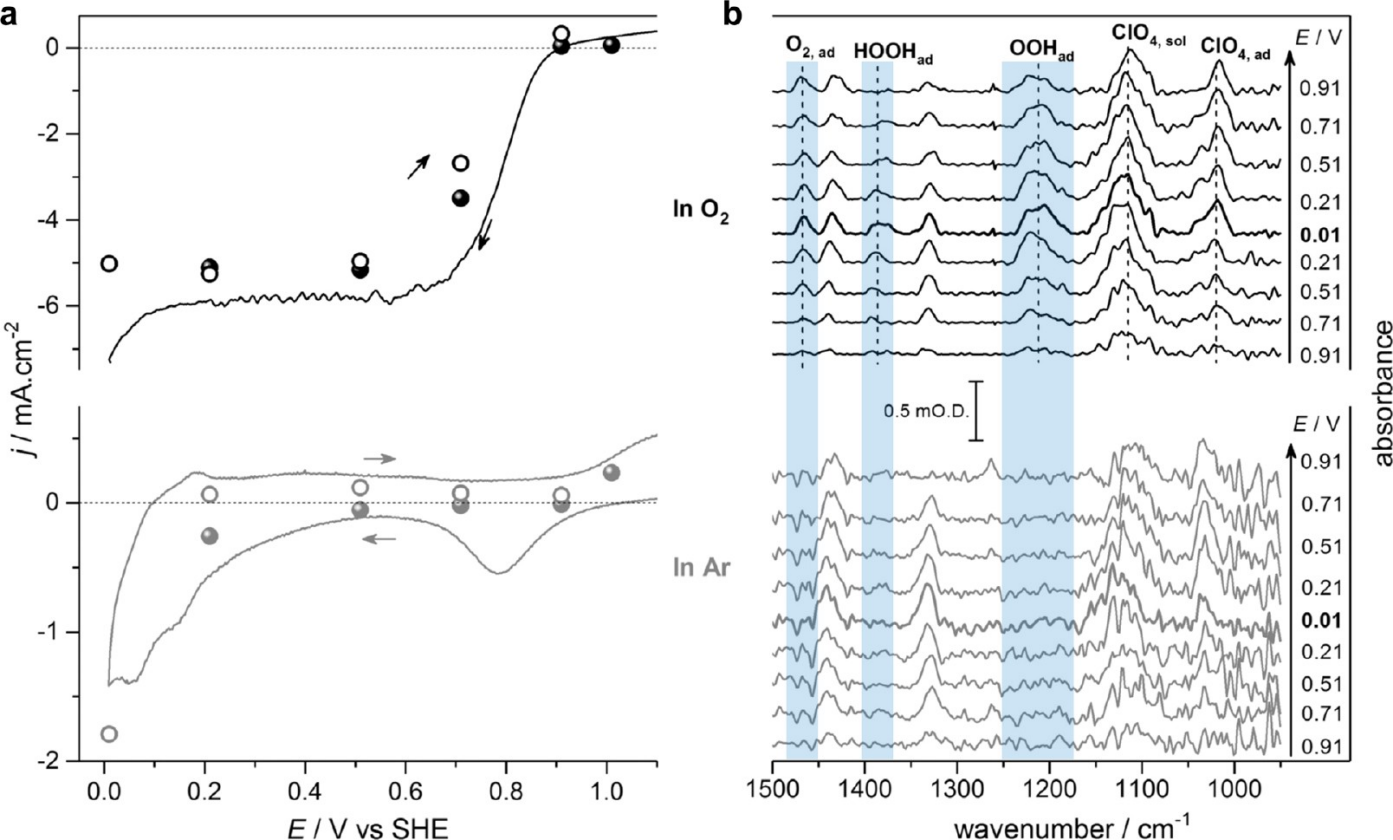
(a) Comparison of voltammetry (lines, scan rate 2 mV s^–1^) and average current densities (*j*: ●, descending potential steps; ○, ascending potential steps) from constant-potential application at Pt/C in O_2_ (black)- and Ar (gray)-saturated 0.1 M HClO_4_ solutions. (b) *In situ* ATR-IR spectra recorded during the constant potential steps shown in (a). Modified and reproduced with permission from ref (133), 2018, Wiley.

Nayak et al.^133^ performed potential-dependent multibounce attenuated total reflection IR (ATR-IR) spectroscopy on Pt nanoparticles. Interaction between ORR intermediates and the Pt nanoparticle surface was investigated by monitoring the stretching frequency of adsorbed molecules. The ORR polarization curve and cyclic voltammogram under Ar atmosphere are displayed in Figure 19a. In the O_2_-saturated solution, three distinct peaks are observed at around 1212, 1386, and 1486 cm^–1^, corresponding to the O–O stretching mode of adsorbed superoxide (*OOH), the OOH bending mode of adsorbed hyperoxide (*HOOH), and the O–O stretching mode of weakly adsorbed molecular oxygen (*O_2_), respectively (Figure 19b). Recently, Xu et al.^134^ studied the promotion mechanism of Pt_3_Co intermetallic nanoparticles for the oxygen reduction reaction using attenuated total reflection surface enhanced infrared absorption spectroscopy (ATR-SEIRAS). The author suggested blue shifts of *O_2_ frequency on the alloy catalysts compared to the pure Pt, demonstrating the decrease of adsorption energy on the Pt surface due to the ligand and strain effects induced by introduction of Co atoms into the Pt lattice.^134^ Spectroscopic techniques play a crucial role in providing vital information regarding the complex interaction between reaction intermediates and the dynamic surfaces structure in electrocatalysis. Despite their significance, exploration of alloy systems for the ORR remains relatively sparse. Further research efforts are required to understand the role of transition metals on Pt-based alloy systems, which in turn will facilitate the development of cutting-edge catalyst designs.

## 5 Conclusions

Many advances in Pt–M alloys for the ORR have been made in the last half-century, with unprecedented increases in activity. In general, three effects are thought to be responsible for the activity enhancement brought about by transition-metal alloys: ligand (or electronic structure) effects, strain (or geometric) effects, and ensemble effects. Ligand effects refer to the influence of nearby atoms on the electronic structure, while strain effects rely on lattice compression or expansion to influence the activity of the surface. Ensemble effects require coordinated and dissimilar clusters to serve a unique function to modify surface activity through a specific arrangement or set of elements; the interplay between these competing and synergetic effects has been the subject of this review.

There is strong evidence demonstrating that ligand effects alter the adsorption characteristics of catalytic surfaces, whereas strain effects have some supporting evidence to explain observations on Pt–M alloys. In addition, there exists scant evidence to suggest that ensemble effects are able to adequately explain the observed phenomena. d-band theory has provided insight into the unique character of ligand and strain effects, providing some guidance toward intelligent material design. The highest activity for the ORR has been achieved with near-surface alloys and bimetallic single-crystal surfaces, providing the strongest evidence for the influence of the ligand effect over catalytic activity, correlating with computational investigations. Though DFT calculations can approximate practical observations, there remains a significant discrepancy between theory and experiment. Experimental techniques have been introduced which provide insight into dissolution characteristics and the role a transition metal may play in altering the stability of nanoparticles and modifiers under electrochemical conditions.
